# Protocol for a feasibility study evaluating a supported self-management intervention for stroke survivors with aphasia (StarStep study)

**DOI:** 10.1186/s40814-024-01589-y

**Published:** 2025-01-30

**Authors:** Faye Wray, Madeline Cruice, Ian Kellar, Anne Forster

**Affiliations:** 1 Academic Unit for Ageing and Stroke Research, Leeds Institute of Health Sciences, University of Leeds, Leeds, LS2 9JT UK; 2 Academic Unit for Ageing and Stroke Research, Bradford Institute for Health Research, Bradford Teaching Hospitals NHS Foundation Trust, Bradford Royal Infirmary, Bradford, BD9 6RJ UK; 3 School of Health and Psychological Sciences, City University of London, Northampton Square, London, EC1V 0HB UK; 4 Department of Psychology, University of Sheffield, Sheffield, S1 2LT UK

**Keywords:** Stroke, Aphasia, Self-management, Feasibility, Qualitative, Quality of life, Complex intervention, Rehabilitation, Speech and language therapy, Speech and language therapists

## Abstract

**Background:**

There is a growing evidence base to support the use of self-management interventions for improving quality of life after stroke. However, stroke survivors with aphasia have been underrepresented in research to date. It is therefore unclear if self-management is an appropriate or effective approach for this group. To address this gap in the evidence base, we have developed a supported self-management intervention (the ‘Living with Aphasia’ intervention) specifically for stroke survivors with aphasia in the first year after stroke. The StarStep study aims to assess the feasibility of implementing and evaluating the intervention (including the feasibility of participant recruitment, the feasibility of delivering facilitator training, the acceptability of the intervention, the fidelity of intervention delivery and outcome data completeness).

**Methods:**

StarStep is a mixed-methods, non-randomised feasibility study. The Living with Aphasia intervention will be facilitated by speech and language therapists and implemented in two community stroke teams in the north of England. We aim to recruit 30 stroke survivors who have aphasia (and/or their family members) and who are ≤ 12-month post-stroke to participate in data collection for the study. Following informed consent, participants will complete a baseline data collection questionnaire which will include measures of quality of life, symptoms of depression and perceived communicative effectiveness. Follow-up questionnaires will be completed at 3-month post-intervention. Qualitative data collection will include implementation groups and semi-structured interviews with speech and language therapists, semi-structured interviews with stroke survivors with aphasia (and/or their family members) and observations of the delivery of the intervention. A joint display table will be used to integrate findings from each element of data collection in order to consider overall feasibility.

**Discussion:**

This study will provide the information necessary to optimise data collection processes and to optimise the implementation and delivery of the self-management intervention. Feasibility data will inform decision-making regarding progression to a future definitive cluster randomised controlled trial to evaluate the effectiveness of the intervention.

**Trial registration:**

ISRCTN registry, trial registration number: ISRCTN10401966. Date of registration: 07/10/2023. URL of trial registry record: https://doi.org/10.1186/ISRCTN10401966.

**Supplementary Information:**

The online version contains supplementary material available at 10.1186/s40814-024-01589-y.

## Background

Stroke remains a leading cause of long-term, complex disability [1, 2]. Around one-third of stroke survivors will experience the language impairment aphasia [3]. Aphasia affects the comprehension or expression of spoken, written or sign language. Aphasia is associated with poor outcomes both in the short term (longer hospital stays, increased risk of mortality and disability at 1-month post-stoke) [3, 4] and in the longer term (reduced quality of life, reduced social participation and increased risk of depression) [5–7]. Qualitative research also suggests the lasting impact of aphasia; with some struggling to overcome barriers to social participation, or, to find a sense of purpose or satisfaction in their daily lives [8].

Self-management interventions are designed to help people with chronic diseases gain the knowledge and skills they need to manage the physical, social and emotional consequences of living with their condition [9–11]. They typically include multiple components such as education, goal-setting, problem-solving, action planning, self-monitoring and decision-making [9–11] and have been delivered in various modes including group-based, telephone-based and individually delivered approaches [12]. Multiple healthcare professionals (e.g. nurses, allied health professionals, psychologists) and lay people have been trained to facilitate self-management interventions [12]. Recently, the term ‘supported self-management’ has been used more frequently, and interchangeably, with the term ‘self-management’. This is to emphasise the importance of support/enablement as part of this approach [13] and to overcome the negative connotations of being left to manage alone which may be associated with the term ‘self-management’ [14].

Self-management interventions are increasingly being developed and tested as a potential pathway for improving longer-term care and outcomes after stroke. Clinical guidelines in a number of countries (including the UK [15, 16], USA [17], Canada [18] and Australia [19]) have recommended that stroke survivors are offered the opportunity to learn self-management skills as part of longer-term care. A Cochrane review suggested benefits of stroke self-management interventions upon quality of life and self-efficacy [10]. However, stroke survivors with aphasia (SSWA) have been underrepresented in research in this area to date [20], and therefore, the suitability and effectiveness of such approaches for this population are unclear.

To address this gap in the evidence base, we have developed a supported self-management intervention specifically for SSWA in the first year after stroke. Medical Research Council guidance [21] was used as a framework for developing the intervention. In line with this framework, the intervention has been developed iteratively, informed by a number of pieces of research including systematic reviews [8, 20], a qualitative needs assessment with key stakeholders (including SSWA, their family members and speech and language therapists) [22, 23] and a co-production study [24]. The development of the intervention has also been informed by behaviour change theory [25]. The Living with Aphasia intervention is a multicomponent approach which is designed to be delivered on a one-to-one basis by speech and language therapists in the community setting. The components of the intervention include the following: an accessible written guide for SSWA, a guide for family and friends, training for speech and language therapists and a toolkit for speech and language therapists to support the integration of the approach in practice. Further details of the intervention are provided in the ‘Methods/design’ section.

This study (the StarStep study) will test the feasibility of implementing the intervention in practice and clarify procedures for a future definitive cluster randomised controlled trial (RCT) (with internal pilot phase) to evaluate the effectiveness of the intervention.

### Study aim

The overarching aim of this study is to assess the feasibility of implementing and evaluating the intervention to inform the design of a future effectiveness RCT.

### Objectives

#### Feasibility objectives

1. To assess the feasibility of *recruitment methods* and uptake by the following:
  a Assessing the number of SSWA screened, identified as eligible and for whom informed consent/consultee declaration (assent) can be obtained.
2. To assess the feasibility of *intervention implementation* and *delivery* by the following:
  a Assessing the feasibility of recruiting and training speech and language therapists (SLTs) to facilitate the intervention.
  b Obtaining an understanding of facilitators’ (SLTs) views of the intervention (including acceptability, barriers and enablers to implementation).
  c Obtaining an understanding of participants’ (SSWA, family members/friends) views of the intervention (including acceptability, unanticipated consequences).
  d Exploring fidelity of intervention delivery (including influencing contextual factors).
  e Explore the costs and resource use associated with delivering the intervention.
3. To assess the feasibility of collecting *outcome measures* including completion rates, levels of missing data and ease of use.

#### Other objectives

4) To *test* and *refine* the proposed theoretical *logic model* for the intervention which maps out intervention components, mechanisms and outcomes (based on established behaviour change theory [25]). This will enable us to develop our understanding of which components of the intervention produce which outcomes and thus a reasoned approach for refining the intervention if needed [26].

## Methods/design

This protocol is reported drawing on SPIRIT guidance [27, 28] and the CONSORT extension for reporting pilot and feasibility trials [29–31]. A SPIRIT checklist is provided in Additional file 1.

### Study design and setting

This study is a mixed-methods, non-randomised, feasibility study. We will implement the intervention in two community speech and language therapy services in the north of England. The intervention will be delivered at the level of the whole service as ultimately a cluster RCT is planned.

To meet the study objectives, qualitative and quantitative data will be collected. Quantitative data collection will include screening data, cost and resource data, and outcome measures. Qualitative data collection will include implementation groups with SLTs, semi-structured interviews (with SSWA, family members, SLTs) and observation of intervention delivery. Further detail is provided in the ‘Data collection methods’ section of this protocol.

### Participants, interventions and outcomes

#### Eligibility criteria

- Stroke survivors with aphasia
  Inclusion criteria
  1. Are aged 16 years or over
  2. Have a primary diagnosis of stroke
  3. Have post-stroke aphasia (as diagnosed by the treating speech and language therapy service)
  4. Are ≤ 12-month post-stroke
  5. Are able and willing to provide informed consent for participating in data collection (e.g. outcome measures or observations or a semi-structured interview) or for whom a consultee declaration (assent) is provided (observations only).
  6. Living at an address within the remit of a participating community service and referred for speech and language therapy from a participating community service.
  Exclusion criteria
  1. > Twelve-month post-stroke
  2. Already in receipt of speech and language therapy from the participating community stroke team
  3. In receipt of end-of-life care (documented in medical notes)
  4. Residents in nursing or care homes
  5. People with comorbid progressive neurological disorders, e.g. Huntington’s disease, motor neuron disease, Parkinson’s disease and multiple sclerosis
- Family members^1^
  Inclusion criteria
  1. Are aged 16 years or over
  2. Are a family member/close friend and/or carer of a SSWA participating in the study or are a family member/close friend and/or carer of a SSWA who lacks capacity to consent to participate in the study
  3. Are a family member/close friend and/or carer who provides help and support (practical and/or emotional) to the SSWA at least once a week
  4. Are able and willing to provide informed consent for participating in data collection
  Exclusion criteria
  1. They are caring for a stroke survivor resident in a nursing or care home or with palliative care needs.
  2. They are caring for a stroke survivor with a comorbid progressive neurological disorder, e.g. Huntington’s disease, motor neuron disease, Parkinson’s disease and multiple sclerosis
- Speech and language therapists
  For SLTs, the following eligibility criteria will apply for participation in implementation groups, observation of intervention delivery and semi-structured interviews:
  Inclusion criteria
  1. Are employed as a SLT at a participating community stroke team
  2. Has a caseload including SSWA
  3. Has received training in facilitating the self-management intervention
  4. Is willing to provide informed consent to participate in the implementation group/observation of intervention delivery/semi-structured interview
  Exclusion criteria
  - N/A

#### Intervention

The provisional self-management intervention (the Living with Aphasia approach) consists of four components.

- Component 1: An accessible guide for SSWA
- Component 2: A guide for family and friends
- Component 3: Training for SLTs
- Component 4: A toolkit for SLTs to support the integration of the approach in practice

SLTs will be provided with 1 day of training in the intervention (component 3). The intervention is designed to be integrated within usual speech and language therapy provision in the community setting. The average number of speech and language therapy sessions provided by any particular community team varies across the country. However, recent national audit data provided by the Sentinel Stroke National Audit Programme (SSNAP) (April-June 2019) [32] suggests that, on average, stroke survivors will receive approximately seven sessions of speech and language therapy in the community setting with a median session time of 47.5 min. Sessions are usually provided individually and face to face. It is expected that during these sessions, SLTs will introduce the guides (components 1 and 2), and, in collaboration with the SSWA and their family member, the SLT will agree goals and weekly activities to be completed outside of session time (component 4). The activities are designed to support and encourage self-management with a particular emphasis on overcoming barriers to social participation and participation in meaningful activity. It is anticipated that family members will be involved in supporting weekly activities (with help and training on how to do this provided by the SLT). Weekly activities will be reviewed at the beginning of subsequent sessions to help with problem-solving and planning of the next activity. At the end of therapy, the SLT will help the SSWA and their family member to make a self-management plan to help them to build upon the skills and strategies they have learnt moving forwards.

A detailed description of the intervention is provided in Table 1. using the Template for Intervention Description and Replication (TIDieR) checklist [33] as a framework.

**Table 1:** Description of the supported self-management intervention for stroke survivors with aphasia

**Intervention name**	A supported self-management intervention for SSWA (the Living with Aphasia approach)
**Why**	Self-management programmes can have a positive impact upon quality of life and reduce healthcare utilisation [10, 34–36]. However, there is a lack of evidence for self-management programmes to support stroke survivors with aphasia [37]. We have developed a self-management programme which is designed specifically to be accessible to stroke survivors with aphasia and to address their needs. The goal of the intervention is to support and enable stroke survivors with aphasia to self-manage; focusing on increasing social participation and participation in meaningful activity. A logic model for the intervention has been developed (based on behaviour change theory (25)) which maps out the proposed inputs, mechanisms of action and outcomes for each component of the intervention (accessible guide for SSWA, a guide for family and friends, training for SLTs and a toolkit for SLTs).
**What: Intervention components and materials**	*Component 1*: An accessible guide for SSWA. SSWA will be provided with a written guide: ‘Living with Aphasia: A guide for stroke survivors’. The guide is formatted according to the Stroke Association’s guidelines for accessible information [38]. The guide contains short and simple information about the following:Self-management and its health benefits, Practical and alternative communication strategies, Strategies to cope with low mood and fatigue. The guide contains sections for the stroke survivor with aphasia to fill in during therapy to aid goal setting and planning for the end of therapy. The guide contains stories from other SSWA about how they manage
	*Component 2*: A guide for family and friends. A family member or friend will be provided with a written guide ‘Living with Aphasia: A guide for family and friends’. The guide contains short and simple information about the following:What speech and language therapy is and what can be expected in sessions, Strategies to communicate with someone with aphasia, Strategies to cope with the increased demands of supporting their relative with aphasia, Where to go for support for themselves and their relative (national and local organisations). The guide also contains a section for the family member/friend to fill in to help with troubleshooting difficulties with communication. There is also space for personalised information to be provided by the speech and language therapist
	*Component 3*: Training for speech and language therapists. Training for speech and language therapists will be provided to help them to identify and support opportunities to support self-management within therapy. The training includes the following:Education about self-management and the health benefits of this for SSWA and their family/friends, Communication skills training to support self-management within the therapeutic relationship (including practical demonstrations and role play), Training in implementing the toolkit (component four) within therapy sessions, tailoring use to the individual, Examples of opportunities to support self-management within speech and language therapy
	*Component 4*: A toolkit for speech and language therapists to support the integration of the self-management approach in practice. The toolkit consists of written worksheets/activities to be completed with SSWA and/or family/friends and written instructions for speech and language therapists about how to use the worksheets/activities in therapy. The toolkit includes the following:A standardised approach to setting participation goals and actions, Activities to help SSWA develop a routine and take part in meaningful activity, Activities to help SSWA to build or maintain support networks
**What: Intervention procedures**	SSWA will be identified by treating speech and language therapy services in the acute or community setting. The guides (components 1 and 2) will be introduced by the treating speech and language therapist who will reinforce information about self-management and its health benefits and what to expect during therapy. The stroke survivor with aphasia (and family member/friend as appropriate) will set a goal for participation jointly with the speech and language therapist. Depending upon the goal, the speech and language therapist will tailor activities from the toolkit (component 4) throughout the remainder of therapy sessions. SSWA will plan a weekly activity to be completed outside of session time. This will be reviewed at the beginning of each session to help with problem-solving and motivation and to plan next week’s activity. At the end of therapy, the speech and language therapist will help the stroke survivor with aphasia (and family member/friend as appropriate) to reflect on their progress and devise a ‘self-management plan’ (written within their guide [component 1]). The plan includes information about the skills and strategies the person with aphasia has learnt during therapy and what they would like to continue to work on moving forwards
**Who provided**	The intervention will be facilitated by qualified speech and language therapists in the community setting whose caseloads include SSWA. The training for speech and language therapists will be co-facilitated by the chief investigator (who has disciplinary expertise in psychology and self-management) and a qualified clinical neuropsychologist who is experienced in delivering clinical communication skills training. The clinical neuropsychologist is not a member of the research team or involved in any other research proccesses. The training will be provided over 1 day at the community site. Supervision of staff delivering and supporting the intervention will be by usual NHS line management
**How**	The intervention will be delivered face to face by speech and language therapists on an individual basis. Speech and language therapists will work with the person with aphasia and/or their family/friend to find ways to encourage and support self-management. This support will be graded, and the person with aphasia and/or their family/friend will be encourage to take the lead with planning and undertaking activities to support self-management gradually as the sessions progress
**Where**	The intervention will be delivered in the community setting by community stroke teams and/or early supported discharge teams. Most frequently, therapy sessions will be held in the participant’s home. However, occasionally, therapy sessions may be held at an outpatient clinic
**When and how much**	The intervention is designed to be integrated within usual speech and language therapy provision in the community setting. It is anticipated that community services involved in the study will provide a similar number of sessions to the national average of seven sessions with a median session time of 47.5 min. The period of time over which the sessions will be delivered is anticipated to be between 6 and 12 weeks
**Tailoring**	Personalised information will be provided by the speech and language therapist, prompted by use of the guides (components 1 and 2). Personalised individual goals and weekly activities (component 4) will be agreed to support the person with aphasia to self-manage. Factors which may influence the personalisation of goals and weekly activities include the severity of aphasia, availability of support from family or friends and the participant’s level of confidence. The speech and language therapist will use their clinical expertise to tailor weekly activities and provide support accordingly
**Modifications**	Modifications will be considered on an ongoing basis during the course of the feasibility study. Any modification made will be logged in a table of change [39] which records the comments which prompted a change, what the proposed change is and what change was discussed agreed with the wider research team
**How well**	Fidelity to the intervention will be monitored as part of the ongoing feasibility study based on Carroll et al.’s [40] framework. Carroll et al. define fidelity in terms of adherence (to planned intervention content, frequency/duration of sessions and whether the intervention reached intended participants) and moderating factors (participant responsiveness, quality of delivery, facilitation strategies). We will use multiple sources of data (including interview, observational, quantitative and questionnaire data) to evaluate fidelity

#### Sample size

As this is a feasibility study, a power calculation is not appropriate. We aim to recruit 30 SSWA and/or family members to participate in this study. SSWA may participate independently, or a family member may also consent to participate in the study with the person with aphasia (dyad). A family member may participate in the study independently if their relative with aphasia lacks capacity to consent; however, family members will not be eligible to participate if their relative has capacity but does not wish to participate in the study. Therefore, the sample size of 30 will be made up of both independent participants (SSWA, family members of SSWA who lack the capacity to consent) and dyads (SSWA and a family member/friend). Dyads will count as one recruit towards the total sample size of 30. From the total sample of 30 participants, we aim to purposively sample between 10 and 15 SSWA and/or family members to take part in the in-depth semi-structured interviews and observations. We will invite all SLTs who are trained in the intervention to take part in an in-depth semi-structured interview (*n* = 10–15). These sample sizes will enable the aims/objectives of this project to be fulfilled and to obtain the data necessary to inform a future effectiveness cluster RCT.

#### Recruitment

We propose to identify and recruit SSWA and/or family members in two ways:

##### In the hospital setting

All inpatients within participating acute services will be screened for eligibility by experienced and appropriately trained Clinical Research Network (CRN) practitioners or local research staff (e.g. research nurses). CRN practitioners/local research staff will work closely with clinical staff at the service (in particular, SLTs) to identify potential study participants who fulfil the eligibility criteria. Once permission to approach has been obtained from the clinical team, CRN practitioners/local research staff will make the first contact with potentially eligible participants and seek informed consent.

##### From the caseloads of community stroke teams

Potential participants will also be identified through screening the caseloads of community stroke teams. A SLT from the community stroke team will be responsible for screening based on the eligibility criteria. Potentially eligible participants will be contacted by a SLT from the community stroke team by telephone or in person (depending upon the severity of the person’s aphasia). The SLT will briefly explain the study and ask the SSWA/family member if they will consider participating. If the potential participant shows an interest, verbal consent may be taken for their contact details to be passed to the research team to discuss their participation further. Or, if the potential participant prefers to receive further information by post, they can return a consent to contact form to the research team using a freepost envelope which will be provided. As it may be difficult for potential participants with aphasia to return a consent to contact form (e.g. due to difficulties with reading and writing, mobility difficulties or a lack of family member support), a SLT or administrator from the team may conduct one follow-up telephone call to participants who have received information by post to check the invitation has been received and see if the person may be interested in participating. Following receipt of the consent to contact form, the chief investigator (CI) (first author F. W.) will contact potentially eligible participants and seek informed consent.

We will work with service managers at participating community sites to identify SLTs to participate in the implementation groups, observation of intervention delivery and semi-structured interviews.

#### Participant timeline

Table 2 outlines activities and assessments for SSWA and/or family members/friends.

**Table 2:** Participant timeline

Activity/assessment	Pre-study Screening/consent (week -1)	Baseline (week 0)	Intervention (weeks 1–12)	Semi-structured interviews (end of intervention + 2 weeks)	Three-month follow-up (end of intervention + 3 months)
**Enrolment**					
Screening log	X				
Eligibility screen	X				
Informed consent	X				
Demographic data		X			
**Intervention**					
Supported self-management intervention			X		
**Qualitative data collection**					
Observation of intervention delivery			X		
Semi-structured interviews				X	
**Outcome measures**					
Stroke and Aphasia-Quality-of-Life scale (SA-QOL-39)		X			X
Patient Health Questionnaire-8 (PHQ-8)		X			X
Communicative Outcomes after Stroke (COAST)		X			X
Carer COAST (family/friends of SSWA only)		X			X
Caregiver Strain Index (family/friends of SSWA only)		X			X
Stroke Aphasic Depression Questionnaire (SADQ-21) (family/friends of SSWA only)		X			X

#### Data collection methods

##### Feasibility objective 1 (To assess the feasibility of recruitment methods and uptake)

To assess the feasibility of participant recruitment, recruiting sites (local research staff) will also be asked to record anonymised screening data for all SSWA (e.g. age, gender, reasons for ineligibility) and those for whom consent/consultee declaration (assent) can be obtained.

##### Feasibility objective 2 (To assess the feasibility of intervention implementation and delivery)

A number of data collection methods will be used to assess the feasibility of intervention implementation and delivery. To meet objective 2a, we will collect data on the number of SLTs attending the training and the number of SLTs eligible to receive the training in the service. To meet objective 2e, cost and resource data use will be collected for each participant (e.g. number of sessions, SLT travel and preparation time) by an SLT completed log, and we will also work with sites to collect service level data (e.g. type of service, staffing levels, average number of sessions for usual care) to inform out analysis of intervention costs. To meet objectives 2b, c and d, we will collect qualitative data using a number of methods:

##### Implementation groups (objectives 2b, 2d)

We will establish an implementation group at each of the participating services to support the implementation of the intervention and to understand acceptability, barriers and enablers to implementation. The groups will involve SLTs who have been trained to deliver the intervention, service managers/clinical leads and a member of the research team. The first implementation group will be scheduled approximately 2 weeks after the training. Subsequent implementation groups will be held throughout the recruitment period, on a monthly basis, approximately. Frequency of meetings may differ between the services reflecting the local situation. The group will discuss, review and refine implementation strategies for the intervention [41]. Appropriate strategies will be developed with clinical teams to reflect individual service contexts. Action plans and agreed timelines will be developed. Obstacles encountered in implementation and solutions developed will also be recorded. With the members’ consent, we will make a written and audio recording of the meetings and develop a pro-forma for capturing decision-making and action plans from each meeting to aid our understanding of the implementation process. Audio recordings of the meetings will be used as part of qualitative data analysis (see the ‘Data analysis’ section for further details).

##### Semi-structured interviews (objectives 2b, 2c)

All semi-structured interviews will be undertaken by the CI (F. W.). Where possible, interviews will be conducted face to face; however, following COVID-19, we will also make contingency plans for interviewing participants remotely (by video call or telephone). Separate informed consent will be obtained for this aspect of the study. All interviews will be audio recorded.

##### Stroke survivors with aphasia

A purposive sample of SSWA will be recruited to take part in a semi-structured interview. The interviews will aim to explore participant’s views of the intervention. We will aim to sample stroke survivors to represent different severities of aphasia (mild, moderate, severe) and living circumstances (alone/with others). The interviews will be scheduled 2 weeks after participants have finished receiving the intervention.

 To support the participation of SSWA in the interview process, a number of strategies will be used. Supported conversation techniques [42–44] will be used to maximise communication, for example writing key words down, using pictures, simplifying language and/or using communication boards. FW has experience of interviewing SSWA and has received ‘active communication’ training from the Stroke Association. The option to have a family member or other communication partner present at interviews will be available to all SSWA who wish to participate.

##### Family members

A purposive sample of family members will be recruited to take part in a semi-structured interview. We will aim to sample family members to represent people caring for stroke survivors with different severities of aphasia (mild, moderate, severe) and different relationship types (e.g. spouses, friends, other caregivers). The interviews will be scheduled 2 weeks after the intervention has finished. The interviews will aim to explore participant’s views of the intervention. SSWA and family members/friends will be given the option to be interviewed as a dyad or separately. If interviewed separately, the researcher will discuss if the interviews should be arranged on separate days or the same day. The researcher will follow participant’s wishes and arrange a joint or separate interviews accordingly. Where interviews are conducted together, the researcher will be mindful to ensure that the SSWA is supported to express their views throughout the interview [45, 46], e.g. by being clear at the beginning of the interview that some questions will be directed towards the SSWA and some for the family member and by clarifying throughout where views/experiences are shared or where they are different.

##### Speech and language therapists

All staff who have received training in the intervention will be invited to participate in a semi-structured interview. The interviews will aim to explore SLT’s views of the intervention (including acceptability, facilitators and barriers to implementation). The interviews will take place at a convenient location (usually the SLT’s place of work) once recruitment for the study has ended.

##### Observation of intervention delivery (objective 2d)

To gain insight into the fidelity of intervention delivery, we will test the feasibility of observing intervention delivery in two ways: (1) video recording and (2) researcher observation. Permission for video recording will be obtained during the consent process, and ongoing consent will be checked at the beginning of each session before recording begins. If during the early stages of the study it becomes apparent that it is unfeasible to video record therapy sessions, researcher observations of intervention delivery will be undertaken. Variation in approach may also depend upon COVID-19 restrictions. All researcher observations will be undertaken by the CI (F. W.) who will shadow SLTs as they carry out home visits or outpatient clinic sessions with recruited participants. The timings and locations of community-based observations will be negotiated with relevant staff in the service. We will aim to observe one session per recruited participant and to sample a range of session types (i.e. at the beginning, middle and end of the intervention). A qualitative observational framework will be used to record delivery of intervention components.

##### Feasibility objective 3 (To assess the feasibility of collecting outcome measures)

We will assess the feasibility of collecting a number of outcome measures. Measures will be collected at baseline and at 3 months. SSWA will be asked to complete the Stroke and Aphasia Quality-of-Life Scale (SA-QOL-39) [42], Patient Health Questionnaire (PHQ-8) [43, 44] and Communication Outcomes after Stroke (COAST) [47] measures. Family members will be asked to complete the Carer COAST [48], the Modified Caregiver Strain Index [49] and the Stroke Aphasic Depression Questionnaire (SADQ-21) [50]. Where possible, baseline data and outcome measures will be collected face to face by a member of the research team. However, following COVID-19, we will also make contingency plans for collecting outcome data remotely (e.g. by video call)﻿.

In addition to outcome measures, at baseline we will also demographic data from participants. For SSWA, this will include age, gender, ethnicity, time since stroke, occupational status, living circumstances (alone/with others), disability level (Barthel index) [51], level of education, and severity of aphasia (Frenchay Aphasia Screening Test [FAST]) [52]. Where possible, from the SSWA’s medical record, we will record the following: type of stroke, stroke severity, comorbidities and the modified Rankin Scale score. For family members, demographic data collection will include self-reported; age, gender, ethnicity, relationship to the SSWA, level of education and occupational status. We will also ask SSWA to complete a brief therapeutic alliance measure (session rating scale [53]) after each therapy session. These will be collected by SLTs and returned to the research team.

##### Other objectives (To test and refine the proposed theoretical logic model)

No further data collection will be required to meet objective 4. The logic model for the intervention will be tested and refined by the triangulation and secondary analysis of qualitative data (semi-structured interviews, observations, implementation groups) outlined above.

### Data management

All data will be stored securely and analysed at the Academic Unit for Ageing and Stroke Research (a department of the University of Leeds based at Bradford Teaching Hospitals NHS Foundation Trust, UK). Analyses will primarily be undertaken by the CI (F. W.). Members of the wider research team will have access to the anonymised/pseudonymised data only. Data will be stored securely for 3 years after the study has ended and then destroyed.

### Data analysis

#### Feasibility objective 1 (To assess the feasibility of recruitment methods and uptake)

To assess the feasibility of recruitment, data on the number of potential participants screened, number approached and number eligible and recruited will be summarised overall, by month and by site.

#### Feasibility objective 2 (To assess the feasibility of intervention implementation and delivery)

The feasibility of recruiting and training speech and language therapists to facilitate the intervention (objective 2a) will be assessed using training data (number of therapists trained compared to the number of therapists eligible for training within the service). To explore the cost and resource use associated with the intervention (objective 2e), we will compare the average cost of the sessions received and therapy time versus usual care within the service.

Qualitative data analysis will be used to assess study objectives 2b and c. Qualitative data (semi-structured interviews, observations, implementation groups) will be used to explore the acceptability of the intervention and barriers and facilitators to implementation. Separate analyses will be conducted for data collected from SSWA/family members/friends and data collected from SLTs. Written transcripts of audio recordings from semi-structured interviews and implementation groups will be checked for accuracy prior to analysis. Data will be analysed using framework analysis [54]. Framework analysis is a systematic and comprehensive approach to qualitative data analysis which is based upon the use of a coding framework developed specifically for the data being analysed. To create the initial coding framework, Ritchie et al. [54] not only encourage familiarisation with the data itself (i.e. transcripts, field notes) but also draw upon the existing literature (e.g. previous research or theoretical frameworks). We will draw upon Sekhon et al.’s [55] framework to explore acceptability within all interviews, and to explore barriers and facilitators to implementation for SLTs, we will draw upon the Consolidated framework for Implementation Research [56] (a framework for assessing contextual barriers or facilitators to implementation) and normalisation process theory [57] (a framework for exploring factors which affect the routine implementation of interventions into practice). Concepts from these frameworks may be used directly in the coding framework or may inform the generation of bespoke codes (as appropriate to the data collected).

Fidelity to the planned intervention (objective 2d) will be assessed using a number of sources of data. We will draw upon Carroll et al.’s [40] framework which defines fidelity in terms of adherence (to planned intervention content, frequency/duration of sessions and whether the intervention reached intended participants) and moderating factors (participant responsiveness, quality of delivery, facilitation strategies). To evaluate adherence to planned intervention content, we will analyse observational data (video recordings and/or researcher observations). We will develop a proforma to evaluate the presence or absence of expected intervention content (based upon the intervention manual). Quantitative data (cost and resource data/anonymised screening data) will be used to evaluate frequency/duration of sessions and whether the intervention reached intended participants. We will also consider moderating factors in our analysis of fidelity. To explore the quality of delivery, we will develop a proforma (based upon similar checklists [58]) to evaluate the presence or absence of a therapeutic alliance in therapy sessions using observational data (video recordings and/or researcher observations), and we will also summarise questionnaire data from the session rating scales completed by SSWA. To explore participant responsiveness and facilitation strategies, we will produce a narrative summary by drawing upon the qualitative analysis of interview data and data from implementation groups. A summary of questionnaire data collected at the end of training (to assess SLTs understanding of the intervention) will also be used in the narrative summary of facilitation strategies.

#### Feasibility objective 3 (To assess the feasibility of collecting outcome measures)

The feasibility of collecting outcome measures will be assessed by summarising the number of outcome assessments completed, completion rates of questionnaires within the assessments and missing data (at the participant and item level) at baseline and 3-month follow-up. As this is a feasibility study, the analysis of outcome measures will not involve hypothesis testing and will be descriptive in nature. Descriptive statistics (e.g. mean, standard deviation [SD], range) will be used to summarise total scores for the outcome measures at baseline and follow-up.

#### Other objectives (To test and refine the proposed theoretical logic model)

The logic model for the intervention (objective 4) will be tested and refined by the triangulation and secondary analysis of qualitative data (semi-structured interviews, observations, implementation groups). We will use the meta-matrix method [59–61] to explore the extent to which themes developed from the initial analysis of interviews, observations and implementation groups are related to the theoretical constructs in the logic model. The logic model details the planned:

- Inputs (intervention components)
- Activities (behaviour change techniques targeted by each intervention component)
- Outputs (theoretical constructs targeted defined by the theoretical domains framework)
- Short-term effects (increased communicative confidence, social participation, participation in meaningful activity and reduced symptoms of low mood for SSWA)
- Long-term effects (improved quality of life, increased perceived communicative effectiveness in everyday situations and lower rates of depression for SSWA and reduced caregiver strain for family/friends).

Themes from interviews, observations and implementation groups will form the ‘rows’ of the matrix, and theoretical constructs will form the ‘columns’. Relevant supporting data will be used to populate the matrix and allow for the logic model to be refined. Findings from the analysis will also be used to generate hypotheses which can be tested quantitatively in a definitive RCT.

### Criteria for continuation to a definitive RCT

Progression criteria for continuation to a definitive RCT (with internal pilot phase) are based on our feasibility objectives and include criteria relating to participant recruitment, staff training, participant retention, intervention implementation and outcome data completeness. The criteria are detailed in Table 3

**Table 3:** Progression criteria for continuation to a definitive RCT

Criteria	Green (proceed with RCT with internal pilot phase)	Amber (proceed with changes)	Red (do not proceed unless changes are possible)
1. **Feasibility of participant recruitment** Can stroke survivors with aphasia be recruited?	≥ 80% of target (*n* = 30) recruited	≥ 70–79% of target (*n* = 30) recruited	< 69% of target (*n* = 30) recruited
2. **Feasibility of staff training** Can SLTs at participating sites be trained in the intervention?	> 80% of eligible staff receive training	≤ 79–70% of eligible SLT staff receive training	< 70% of eligible SLT staff receive training
3. **Feasibility of participant retention** Can outcome assessments be completed at 3 months?	≥ 75% of outcome assessments are completed	< 75 but ≥ 60% of outcome assessments are completed	< 60% of outcome assessments are completed
4. **Intervention implementation** Are key components of the intervention delivered to participants?	An average of three out of four key components are delivered to participants	An average of two out of four key components are delivered to participants	An average of one or less of the four key components are delivered to participants
5. **Intervention implementation** Is the intervention acceptable to to participants (stroke survivors with aphasia and their family members)?	Intervention is identified to be highly acceptable by qualitative data with few issues identified	Intervention is identified to be acceptable by qualitative data with some nonserious^a^ issues identified	Intervention is identified to be of low acceptability with serious^b^ issues identified
6. **Intervention implementation** Is the intervention acceptable to facilitators (SLTs)?	Intervention is identified to be highly acceptable by qualitative data with few issues identified	Intervention is identified to be acceptable by qualitative data with some nonserious^a^ issues identified	Intervention is identified to be of low acceptability with serious issues^b^ identified
7. **Data completeness** Are all items within outcome measures completed?	≥ 75% of items are completed	< 75 but ≥ 60% of items are completed	< 60% of items are completed

^a^Nonserious issues may include those which can be rectified before a definitive RCT such as reformatting of written materials, addition of further detail to the SLT training and addition of strategies to support implementation

^b^Serious issues may include those which cannot be rectified before a definitive RCT such as the intervention being overly burdensome to participants or flaws with the intervention which make it unlikely it would be implemented by SLTs or clinical services

The criteria are based on the traffic light red (do not proceed unless changes are possible), amber (proceed with changes) and green (proceed with RCT with internal pilot phase) system. The criteria were developed in collaboration with our project management and patient and public involvement (PPI) group. A joint display Table [62] will be created to summarise key findings from each element of data collection according to these criteria. This will enable the research team to consider the evidence for each domain, explore areas of agreement or dissonance in the data collected and integrate the findings of the study to consider overall feasibility. The progression criteria will be treated as guidelines rather than rules [63], and we will consult with our project management and PPI group, considering the criteria as a whole, before deciding whether the intervention should continue to RCT testing.

### Data monitoring

#### Harms

In the stroke survivor population, acute illness resulting in hospitalisation, new medical problems and deterioration of existing medical problems are expected. In recognition of this, events fulfilling the definition of an adverse event or serious adverse event will not be reportable in this study with two exceptions. The first exception is death, or, falls or fractures resulting in hospitalization. As these events are expected within the study population, they will not be subject to expedited reporting to the main research ethics committee (REC) but will be reported annually to the REC (in routine annual progress reports) and reviewed by the project management group. The second exception is if the event fulfils the definition of a related and unexpected serious adverse event (RUSAE). All RUSAEs will be reviewed by the CI and reported to the study sponsor (within one working day of the CI becoming aware of the event) and to the REC (within 15 days of the CI becoming aware of the event).

#### Study organisational structure

As defined by the NHS Research Governance Framework, the CI (F. W.) is responsible for the design, management and reporting of the study. The University of Leeds will act as sponsor for the research, and they will delegate responsibilities to the CI and NHS organisations. The sponsors representative can be contacted by emailing governance-ethics@leeds.ac.uk. FW will oversee day-to-day operational conduct of the study. Supervision will be provided on a monthly basis (or as necessary) by co-authors A. F. and M. C. A separate data monitoring committee is not required for a feasibility study of this type (of short duration, where the intervention is of low risk of causing physical or psychological harm to participants and where outcome data is not being used to assess the effectiveness of the intervention). General oversight, including management of risk, will be by the project management group (PMG) which includes co-applicants and expert academics. The PMG will meet at the beginning of the study and every 2 to 3 months thereafter.

### Ethics and dissemination

#### Research ethics approval

Approval for this protocol was granted by the East of England-Essex Research Ethics Committee (Ref.: 21/EE/0115) on 10th June 2021. The protocol reported in this paper is version 2.0 (dated: 24 May 2021). Amendments to the protocol will be approved by the REC and communicated as necessary to participating organisations and participants.

#### Consent/assent

Table 4 shows a summary of the level of consent which will be sought for each element of data collection.

**Table 4:** Summary of level of consent

	Informed consent	Consultee declaration (assent)
Implementation groups		
Speech and language therapists	X	
Quantitative data collection		
SSWA	X	
Family member/friend	X	
Speech and language therapists	X	
Observations of intervention delivery		
SSWA	X	X
Family member/friend	X	
Speech and language therapists	X	
Semi-structured interviews		
SSWA	X	
Family member/friend	X	
Speech and language therapists	X	

Additional consent procedures will be used with SSWA to establish capacity to consent and ensure that consent is voluntary and fully informed. CRN staff/local research staff will be trained in the Mental Capacity Act (MCA) and safeguarding vulnerable adults and will have experience of obtaining informed consent with SSWA. To support discussions about participation in the study, a consent support tool [64] will be used to identify each individual’s language skills and areas of impairment to support their decision-making. Participant information sheets and consent forms have been formatted in different styles according to the recommendations of the consent support tool and using templates created with the National Institute for Health Research (NIHR) to support the inclusion of people with aphasia in research [65]. Examples of accessible information sheets and consent forms are provided in Additional files 2 and 3. In addition to using the consent support tool, the CRN staff/local research staff/FW will engage the potential participant in discussion to assess their ability to retain information and capacity to consent to the study (ask the individual to briefly explain the study back to them or ask forced-choice questions to assess understanding). Where appropriate, the CRN staff/local research staff/research fellow will also engage with treating SLTs or family members/friends in discussions to determine whether they are likely to be able to participate in the study. The CRN staff/local research staff/research fellow will remain responsible for determining whether the stroke survivor can give informed consent to participate in the study.

All SSWA will be assumed to have capacity to consent unless it is established that they lack capacity to consent. Potential participants that lack capacity will only be approached if a ‘supporting carer’ can be identified to support the delivery of the study intervention. In such cases, the ‘supporting carer’ or someone who knows the person’s wishes will be asked to act as consultee and provide consultee declaration (assent) for observations of intervention delivery. Potential participants and consultees will have as long as is needed after receipt of the information to make their decision.

#### Withdrawal

Those approached to participate in this study will have the right to refuse consent/to provide consultee declaration (assent) without having to provide a reason. Those who consent/provide consultee declaration (assent) will be free to withdraw at any time without having to provide a reason. If participants of the proposed study withdraw consent from further participation, their data collected up to that point will be included in the final study analysis. This will be made clear to the participants at the time of consent and when they withdraw from the study.

#### Potentially sensitive information

Discussions within semi-structured interviews are unlikely to cover topics that SSWA, family members or SLTs will find distressing. Nonetheless, this is a possibility as stroke survivors’ and family members’ personal experiences of stroke and life after stroke are likely to be recounted. Participants will be made aware of this prior to consenting to take part. During the interviews, it will be made clear that participants may pause, take a break and/or leave the session at any time. The CI will advise on where SSWA and/or family members may seek further assistance where required, e.g. patient advice and liaison services or the Stroke Association. For SLTs, referral to line managers or staff support resources in their own organisations will be considered.

#### Confidentiality

Identifiable participant data will be securely stored, separate from non-identifiable data at the Academic Unit for Ageing and Stroke Research in Bradford, England. Researcher field notes and audio recordings will be transcribed, and any identifiable participant information will be removed. Direct quotations from participants may be published in research reports and academic journal articles; however, pseudonyms will be used with direct quotations and identifiable information such as name, sites where the study was conducted, and addresses, and dates of birth will be removed and not be published. Transcription will be completed by a professional transcribing company, with audio files and transcripts exchanged via a secure transfer system.

#### Dissemination policy

Outputs from this study will be published in peer-reviewed journals and will also be disseminated at stroke conferences. A lay summary of the results will be placed on the Academic Unit for Ageing and Stroke Research website to facilitate dissemination to the public, professionals and participants. Participants can obtain publications by contacting the CI, as noted in the participant information sheets. Participants will be able to sent an accessible study summary at the end of the project. Results will also be disseminated to the Consumer Research Advisory Group (CRAG) hosted by the Academic Unit for Ageing and Stroke Research (consisting of stroke survivors and their family members) and at consumer conferences wherever possible.

### Patient and public involvement

The CRAG hosted by the Academic Unit for Ageing and Stroke Research has consistently highlighted the need for better long-term support to manage life after stroke. These discussions directly informed the idea for this study. A patient and public involvement (PPI) group consisting of SSWA and their family members helped to develop the fellowship application which provided the funding for this study. SSWA and their family members have also played a fundamental role in the design of the intervention through previous co-production work [24]. A group of SSWA and their family members will continue to advise throughout the research process. For example; reviewing participant information sheets, ethics applications, information sheets, etc. and inputting in to decisions to refine or change the provisional intervention. We will invite a representative(s) from the group to attend PMG meetings. We will work together with the PMG and the group of SSWA/family members to ensure that the results of the study are conveyed to stroke survivors and their family members in an accessible and meaningful way.

## Discussion

Further research is needed to evaluate the effectiveness of self-management approaches as part of longer-term care for SSWA. To address this, we have developed a supported self-management intervention specifically for this population which aims to enable SSWA and their families to develop strategies and confidence to manage life after stroke. This study has been designed to answer critical questions about the feasibility of data collection processes and to understand how best to optimise the implementation and delivery of the intervention. We have taken a mixed-methods approach to enable us to meet our objectives in understanding ‘can this work’ before proceeding to understand ‘does this work’ in a future definitive evaluation [62, 66]. This work will inform the procedures for a future cluster RCT which will ultimately inform the design of longer-term support for SSWA. This study opened to recruitment in May 2023. We anticipate that data collection will cease in August 2024. Results will be submitted for publication from November 2024.

## Supplementary Information

Additional file 1. SPIRIT checklist.

Additional file 2. Accessible information sheet.

Additional file 3. Accessible consent form.

## Data Availability

Not applicable. As this is a small, non-randomised feasibility study, sharing of the dataset generated is not anticipated. However, any requests for data can be made to the corresponding author who will review these on a case-by-case basis with the study team. A data-sharing agreement would be required in this instance.

## Abbreviations

COAST: Communication Outcomes after Stroke scale
CRN: Clinical Research Network
FAST: Frenchay Aphasia Screening Test
PHQ-8: Patient Health Questionnaire-8 item
NIHR: National Institute for Health Research
SADQ-21: Stroke and Aphasia Depression Questionnaire
SA-QOL-39: Stroke and Aphasia-Quality-of-Life questionnaire
SSWA: Stroke survivor with aphasia
SLT: Speech and language therapist
RCT: Randomised controlled trial

## Footnotes

1 Note: Throughout the protocol, we refer to ‘family members’ as the usual significant others who may wish to be involved in the study. However, it may be appropriate for other caregivers (e.g. friends, paid carers) to be involved as a significant other (e.g. where the person with aphasia has no close family member), and we wish to be inclusive in these circumstances.

## References

[1] Johnson CO, Nguyen M, Roth GA, Nichols E, Alam T, Abate D, et al. Global, regional, and national burden of stroke, 1990–2016: a systematic analysis for the Global Burden of Disease study 2016. The Lancet Neurology. 2019;18(5):439–58. 30871944 10.1016/S1474-4422(19)30034-1 PMC6494974

[2] Feigin VL, Forouzanfar MH, Krishnamurthi R, Mensah GA, Connor M, Bennett DA, et al. Global and regional burden of stroke during 1990–2010: findings from the Global Burden of Disease Study 2010. The Lancet. 2014;383(9913):245–55. 10.1016/s0140-6736(13)61953-4 PMC4181600 24449944

[3] Flowers HL, Skoretz SA, Silver FL, Rochon E, Fang J, Flamand-Roze C, et al. Poststroke aphasia frequency, recovery, and outcomes: a systematic review and meta-analysis. Arch Phys Med Rehabil. 2016;97(12):2188–201.e8. 27063364 10.1016/j.apmr.2016.03.006

[4] Ellis C, Simpson AN, Bonilha H, Mauldin PD, Simpson KN. The one-year attributable cost of poststroke aphasia. Stroke. 2012;43(5):1429–31. 22343643 10.1161/STROKEAHA.111.647339 PMC4507407

[5] Cruice M, Worrall L, Hickson L. Quantifying aphasic people’s social lives in the context of non-aphasic peers. Aphasiology. 2006;20(12):1210–25.

[6] Hilari K. The impact of stroke: are people with aphasia different to those without? Disabil Rehabil. 2011;33(3):211–8. 20712416 10.3109/09638288.2010.508829

[7] Kauhanen M-L, Korpelainen J, Hiltunen P, Määttä R, Mononen H, Brusin E, et al. Aphasia, depression, and non-verbal cognitive impairment in ischaemic stroke. Cerebrovasc Dis. 2000;10(6):455–61. 11070376 10.1159/000016107

[8] Wray F, Clarke D. Longer-term needs of stroke survivors with communication difficulties living in the community: a systematic review and thematic synthesis of qualitative studies. BMJ Open. 2017;7(10):e017944. 28988185 10.1136/bmjopen-2017-017944 PMC5640038

[9] Barlow J, Wright C, Sheasby J, Turner A, Hainsworth J. Self-management approaches for people with chronic conditions: a review. Patient Educ Couns. 2002;48(2):177–87. 12401421 10.1016/s0738-3991(02)00032-0

[10] Fryer C, Luker J, McDonnell M, Hillier S. Self management programmes for quality of life in people with stroke. The Cochrane Library. 2016;2016(8):CD010442. 10.1002/14651858.CD010442.pub2 PMC6450423 27545611

[11] Lorig KR, Holman H. Self-management education: history, definition, outcomes, and mechanisms. Ann Behav Med. 2003;26(1):1–7. 12867348 10.1207/S15324796ABM2601_01

[12] Taylor SJ, Pinnock H, Epiphaniou E, Pearce G, Parke HL, Schwappach A, et al. A rapid synthesis of the evidence on interventions supporting self-management for people with long-term conditions: PRISMS–Practical systematic Review of Self-Management Support for long-term conditions. Health Serv Deliv Res. 2014;2(53):1–580. 25642548

[13] Burd H, Hallsworth M, Behavioural Insights Team. Supporting self-management: a guide to enabling behaviour change for health and wellbeing using person- and community-centred approaches 2016 11th June 2019. Available from: https://www.health.org.uk/sites/default/files/RtVSupportingSelfManagement.pdf.

[14] Fletcher S, Kulnik ST, Demain S, Jones FJPo. The problem with self-management: problematising self-management and power using a Foucauldian lens in the context of stroke care and rehabilitation. PLoS One. 2019;14(6):e0218517. 31216337 10.1371/journal.pone.0218517 PMC6584009

[15] Party ISW. National clinical guideline for stroke. 5th ed. London: Royal College of Physicians; 2016.

[16] Department of Health. National Stroke Strategy 2007 13th September 2017. Available from: http://webarchive.nationalarchives.gov.uk/20130105121530, http://www.dh.gov.uk/en/Publicationsandstatistics/Publications/PublicationsPolicyandguidance/dh_081062.

[17] Winstein CJ, Stein J, Arena R, Bates B, Cherney LR, Cramer SC, et al. Guidelines for adult stroke rehabilitation and recovery: a guideline for healthcare professionals from the American Heart Association/American Stroke Association. Stroke. 2016;47(6):e98–169. 27145936 10.1161/STR.0000000000000098

[18] Hebert D, Lindsay MP, McIntyre A, Kirton A, Rumney PG, Bagg S, et al. Canadian stroke best practice recommendations: stroke rehabilitation practice guidelines, update 2015. Int J Stroke. 2016;11(4):459–84. 27079654 10.1177/1747493016643553

[19] Stroke Foundation. (Australian) Clinical Guidelines for Stroke Management 2017 6 August 2018. Available from: https://informme.org.au/en/Guidelines/Clinical-Guidelines-for-Stroke-Management-2017.

[20] Wray F, Clarke D, Forster A. Post-stroke self-management interventions: a systematic review of effectiveness and investigation of the inclusion of stroke survivors with aphasia. Disabil Rehabil. 2018;40(11):1237–51. 28271913 10.1080/09638288.2017.1294206

[21] MRC. Developing and evaluating complex interventions: new guidance 2008 17th July 2015. Available from: www.mrc.ac.uk/documents/pdf/complex-interventions-guidance/.

[22] Wray F, Clarke D, Forster A. How do stroke survivors with communication difficulties manage life after stroke in the first year? A qualitative study. Int J Lang Commun Disord. 2019;54(5):814–27. 31273892 10.1111/1460-6984.12487

[23] Wray F, Clarke D, Forster A. “Guiding them to take responsibility”: exploring UK speech and language therapists’ views of supporting self-management of aphasia. Aphasiology. 2020;34(4):411–30.

[24] Wray F, Clarke D, Cruice M, Forster A. Development of a self-management intervention for stroke survivors with aphasia using co-production and behaviour change theory: an outline of methods and processes. PLoS ONE. 2021;16(11):e0259103. 34813602 10.1371/journal.pone.0259103 PMC8610248

[25] Michie S, Atkins L, West R. The behaviour change wheel: a guide to designing interventions. London: Silverback; 2014.

[26] Mills T, Lawton R, Sheard L. Advancing complexity science in healthcare research: the logic of logic models. BMC Med Res Methodol. 2019;19(1):55. 30871474 10.1186/s12874-019-0701-4 PMC6419426

[27] Chan A-W, Tetzlaff JM, Altman DG, Laupacis A, Gøtzsche PC, Krleža-Jerić K, et al. SPIRIT 2013 statement: defining standard protocol items for clinical trials. Ann Intern Med. 2013;158(3):200–7. 23295957 10.7326/0003-4819-158-3-201302050-00583 PMC5114123

[28] Chan A-W, Tetzlaff JM, Gøtzsche PC, Altman DG, Mann H, Berlin JA, SPIRIT, et al. explanation and elaboration: guidance for protocols of clinical trials. BMJ. 2013;2013:346. 10.1136/bmj.e7586 PMC3541470 23303884

[29] Eldridge S, Chan C, Campbell M. CONSORT 2010 statement: extension to randomised pilot and feasibility trials. Pilot Feasibility Stud. 2016;2(1):726. 10.1186/s40814-016-0105-8 PMC5154046 27965879

[30] Thabane L, Hopewell S, Lancaster GA, Bond CM, Coleman CL, Campbell MJ, et al. Methods and processes for development of a CONSORT extension for reporting pilot randomized controlled trials. Pilot and feasibility studies. 2016;2(1):25. 27965844 10.1186/s40814-016-0065-z PMC5153862

[31] Thabane L, Lancaster G. A guide to the reporting of protocols of pilot and feasibility trials. Pilot and Feasibility Studies. 2019;5(1):37. 30858987 10.1186/s40814-019-0423-8 PMC6393983

[32] Royal College of Physicians. Sentinel Stroke National Audit Programme (SSNAP) National Results Portfolio April-June 20192019 7 April 2020. Available from: https://www.strokeaudit.org/results/Clinical-audit/National-Results.aspx.

[33] Hoffmann TC, Glasziou PP, Boutron I, Milne R, Perera R, Moher D, et al. Better reporting of interventions: template for intervention description and replication (TIDieR) checklist and guide. BMJ. 2014;348: g1687. 24609605 10.1136/bmj.g1687

[34] Deakin T, McShane C, Cade JE, Williams RDRR. Group based training for self-management strategies in people with type 2 diabetes mellitus. Cochrane Database of Syst Rev. 2005;2:CD003417. 10.1002/14651858.CD003417.pub2 15846663

[35] Gibson PG, Powell H, Coughlan J, Wilson A, Abramson M, Haywood P, et al. Self-management education and regular practitioner review for adults with asthma. Cochrane Database Syst Rev. 2002;2002(2):CD0011173. 10.1002/14651858.CD001117 PMC7032643 10796600

[36] Zwerink M, Brusse-Keizer M, van der Valk PD, Zielhuis GA, Monninkhof EM, van der Palen J, et al. Self management for patients with chronic obstructive pulmonary disease. Cochrane Database Syst Rev. 2014;2014(3):CD002990. 24665053 10.1002/14651858.CD002990.pub3 PMC7004246

[37] Wray F, Clarke D, Forster A. Post-stroke self-management interventions: a systematic review of effectiveness and investigation of the inclusion of stroke survivors with aphasia. Disabil Rehabil. 2017;40(11):1237–51. 28271913 10.1080/09638288.2017.1294206

[38] Stroke Association. Accessible Information Guidelines. London: Stroke Association; 2012. Available from: https://www.stroke.org.uk/shop/product/accessible-information-guidelines.

[39] Yardley L, Ainsworth B, Arden-Close E, Muller IJP. studies f. The person-based approach to enhancing the acceptability and feasibility of interventions. 2015;1(1):37. 10.1186/s40814-015-0033-z PMC5153673 27965815

[40] Carroll C, Patterson M, Wood S, Booth A, Rick J. Balain SJIs. A conceptual framework for implementation fidelity. 2007;2(1):40. 10.1186/1748-5908-2-40 PMC2213686 18053122

[41] Reason P, Bradbury H. Handbook of action research. London: Sage; 2006.

[42] Hilari K, Byng S, Lamping DL, Smith SC. Stroke and aphasia quality of life scale-39 (SAQOL-39) evaluation of acceptability, reliability, and validity. Stroke. 2003;34(8):1944–50. 12855827 10.1161/01.STR.0000081987.46660.ED

[43] Kroenke K, Strine TW, Spitzer RL, Williams JB, Berry JT, Mokdad AH. The as a measure of current depression in the general population. J Affective Disord. 2009;114(3):163–73. 10.1016/j.jad.2008.06.026 18752852

[44] Wu Y, Levis B, Riehm KE, Saadat N, Levis AW, Azar M, et al. Equivalency of the diagnostic accuracy of the PHQ-8 and PHQ-9: a systematic review and individual participant data meta-analysis. Psychol Med. 2020;50(8):1368–80. 31298180 10.1017/S0033291719001314 PMC6954991

[45] Croteau C, Le Dorze GJA. Overprotection, “speaking for”, and conversational participation: a study of couples with aphasia. Aphasiology. 2006;20(02–04):327–36.

[46] Croteau C, Vychytil AM, Larfeuil C, Le Dorze G. “Speaking for” behaviours in spouses of people with aphasia: a descriptive study of six couples in an interview situation. Aphasiology. 2004;18(4):291–312.

[47] Long A, Hesketh A, Paszek G, Booth M, Bowen AJCR. Development of a reliable self-report outcome measure for pragmatic trials of communication therapy following stroke: the Communication Outcome after Stroke (COAST) scale. Clin Rehabil. 2008;22(12):1083–94. 19052247 10.1177/0269215508090091

[48] Long A, Hesketh A, Bowen AJCR. Communication outcome after stroke: a new measure of the carer’s perspective. 2009;23(9):846–56. 10.1177/0269215509336055 19482891

[49] Thornton M. Travis SSJTJoGSBPS, Sciences S. Analysis of the reliability of the modified caregiver strain index. 2003;58(2):S127–32. 10.1093/geronb/58.2.s127 12646602

[50] Sutcliffe L. Lincoln NJCr. The assessment of depression in aphasic stroke patients: the development of the Stroke Aphasic Depression Questionnaire. 1998;12(6):506–13. 10.1191/026921598672167702 9869254

[51] Mahoney FI. Functional evaluation: the Barthel index. Md State Med J. 1965;14(2):61–5. 14258950

[52] Enderby P, Wood V, Wade D. Frenchay Aphasia Screening Test (3rd Edition). Bodmin: Stass Publications; 2013.

[53] Duncan BL, Miller SD, Sparks JA, Claud DA, Reynolds LR, Brown J, et al. The session rating scale: preliminary psychometric properties of a “working” alliance measure. J Brief Ther. 2003;3(1):3–12.

[54] Ritchie J, Lewis J, McNaughton Nicholls C, Ormston R. Qualitative research practice: a guide for social sciences students and researchers. 2nd ed. London: SAGE; 2014.

[55] Sekhon M, Cartwright M, Francis JJ. Acceptability of healthcare interventions: an overview of reviews and development of a theoretical framework. BMC Health Serv Res. 2017;17(1):88. 28126032 10.1186/s12913-017-2031-8 PMC5267473

[56] Damschroder LJ, Aron DC, Keith RE, Kirsh SR, Alexander JA. Lowery JCJIs. Fostering implementation of health services research findings into practice: a consolidated framework for advancing implementation science. 2009;4(1):50. 10.1186/1748-5908-4-50 PMC2736161 19664226

[57] May CR, Mair F, Finch T, MacFarlane A, Dowrick C, Treweek S, et al. Development of a theory of implementation and integration: normalization process theory. Implement Sci. 2009;4(1):29. 19460163 10.1186/1748-5908-4-29 PMC2693517

[58] Horvath AO. Greenberg LSJJocp. Development and validation of the Working Alliance Inventory. 1989;36(2):223.

[59] Miles MB, Huberman AM. Qualitative data analysis: an expanded sourcebook. 2nd ed. Thousand Oaks: Sage; 1994.

[60] O’Cathain A, Murphy E, Nicholl JJB. Three techniques for integrating data in mixed methods studies. BMJ. 2010;341:c4587. 20851841 10.1136/bmj.c4587

[61] Wendler MC. Triangulation using a meta-matrix 1. J Adv Nurs. 2001;35(4):521–5. 11529951 10.1046/j.1365-2648.2001.01869.x

[62] Aschbrenner KA, Kruse G, Gallo JJ, Plano Clark VL. Applying mixed methods to pilot feasibility studies to inform intervention trials. Pilot and feasibility studies. 2022;8(1):1–13. 36163045 10.1186/s40814-022-01178-x PMC9511762

[63] Mellor K, Albury C, Dutton SJ, Eldridge S, Hopewell S. Recommendations for progression criteria during external randomised pilot trial design, conduct, analysis and reporting. Pilot and Feasibility Studies. 2023;9(1):59. 37061720 10.1186/s40814-023-01291-5 PMC10105402

[64] Jayes M, Palmer R. Initial evaluation of the Consent Support Tool: a structured procedure to facilitate the inclusion and engagement of people with aphasia in the informed consent process. Int J Speech Lang Pathol. 2014;16(2):159–68. 23826849 10.3109/17549507.2013.795999

[65] Collaboration of Aphasia Triallists. Templates for accessible information sheets and consent forms 2021. Available from: https://www.aphasiatrials.org/templates-for-accessible-information-sheets-and-consent-forms/.

[66] O’Cathain A, Hoddinott P, Lewin S, Thomas KJ, Young B, Adamson J, et al. Maximising the impact of qualitative research in feasibility studies for randomised controlled trials: guidance for researchers. Pilot and feasibility studies. 2015;1(1):1–13. 27965810 10.1186/s40814-015-0026-y PMC5154038

