# Supplementary material for: Protocol for a feasibility study evaluating a supported self-management intervention for stroke survivors with aphasia (StarStep study)
Source: Pilot Feasibility Stud. 2025 Jan 30;11:11. doi: 10.1186/s40814-024-01589-y (PMC11780826; doi:10.1186/s40814-024-01589-y)
Supplement: Supplementary file 2 — Additional file 2. Accessible information sheet. [file 40814_2024_1589_MOESM2_ESM.docx]

**Study title**: Feasibility study of a supported self-management intervention for aphasia (StarStep study)

You are being invited to take part in a research study

**What is the research about?**

| 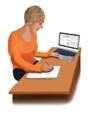 | We are doing some **research.**  It is about improving **support** for **people** with **aphasia.**  Research helps us **learn.**  We need to **know** more about **how to help.** |
| --- | --- |

**Who is doing the research?**

| 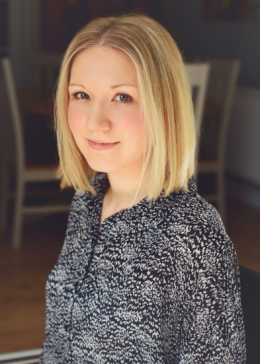  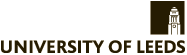 | The **Chief Investigator** is **Dr Faye Wray.**  The research is run from the **University of Leeds.** |
| --- | --- |

**Why are we doing the research?**

| 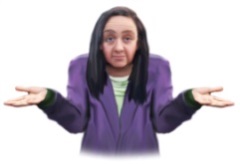 | There is a **new** type of **therapy.**  The **therapy** is to help **build confidence** and **ways** to **cope** with **aphasia**. **Speech and language therapists** give this therapy.  We want to **study** the new **therapy**. We want to see how **feasible** it is to **give** at your local **NHS service**.  You will get the new **therapy** and your **usual therapy**. |
| --- | --- |

**Why me?**

| 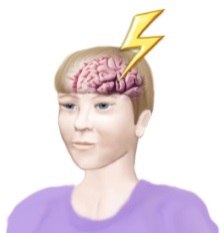 | You have **aphasia** after your **stroke.**  **Aphasia** can make it **hard** to**:**   - Speak - Understand what other people say - Read - Write |
| --- | --- |

**What will I have to do?**

| 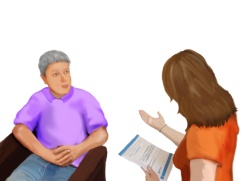 | Do an **assessment**  The **researcher** will **assess** your **language** (when you **join** the **study**). It will take **5-10 minutes**. |
| --- | --- |
| 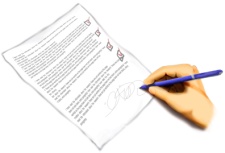 | **Complete** some **questionnaires**  Fill in some **questionnaires** about your **health** (when you **join** the **study** and **6 months** later). It will take **30-60 minutes**.  Fill in a **questionnaire** about the **therapy** after each session. It will take **5 minutes**.  **Tell us** if you need **help**. We will **help you**. |
| **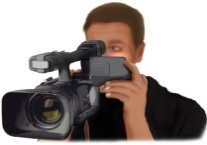**   | **Take part** in **observations**  Your **speech and language therapist** will **video record** your **therapy** sessions.  **Or**  A researcher will **watch one** of your **speech and language**  **therapy** sessions. This will happen if there are any **problems** with the **video recording**.  We would like to **find out** about how the **therapy** works in **practice**. |
| **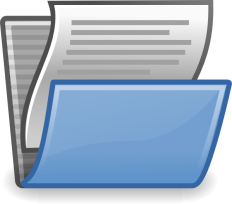** | **Access** to your **medical** and **care records**  We would like to **find out** about;   - The **type** of **stroke** you had. - How your **health** has been **affected**   The information will be **anonymised** so you **cannot** be **identified**. |
|  | **Take part** in an **interview**  We may **contact** you about this. We would like to know your **views** of the **therapy** you **receive**.  You can **agree** or **refuse** at this time. |

**What happens if we can’t meet in person due to the COVID-19 pandemic?**

| 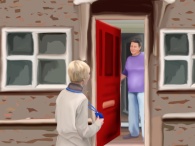 | A researcher may **visit**  you at **home** to:   - complete the **questionnaires** - **watch** a **therapy session** - do an **interview** |
| --- | --- |
| 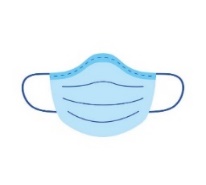 | **But, We** will **not visit** if it is **not safe.**  **We** will **not visit** if you do not **feel comfortable.** |
| 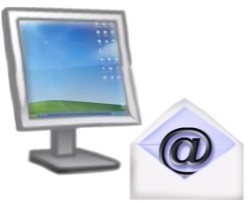 | We may **use** an **on-line platform,** e.g. **Microsoft Teams** or **Skype (**for **questionnaires** and **interviews).** |
| 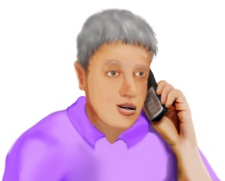 | We will help you to **access** on-line **platforms**.  We will have **regular breaks**. |

**Do I have to take part?**

| 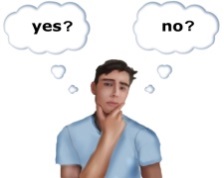 | **You can decide**. You **don't have to.**  If you don’t take part you will **still get your normal help.** |
| --- | --- |
| 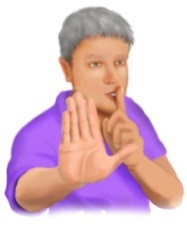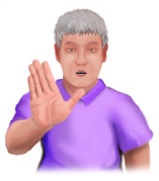 | **You can stop** at any time.  You **don't** have to **give a reason.**  **If you stop** you will still get your **normal help.** |
| 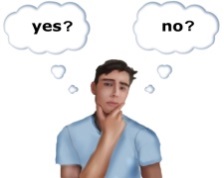 | **You** can **decide**   1. if you want to **take part** in the **observations**. 2. if you want to **complete** the **questionnaires**. 3. If you want to take part in the **observations and** complete the **questionnaires** |

**Who will see the information about me?**

| 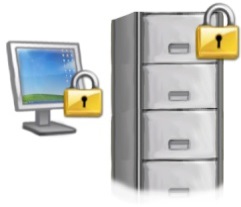 | We will keep the **information** about you **safe.**  **Only** the  **researchers** will see the **information about you.**  We will **keep your information** (at the Academic Unit for Ageing and Stroke Research) **for 3 years.** |
| --- | --- |
| 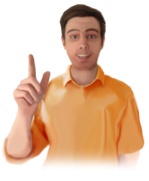 | **But**  if the **researcher is worried** that you or someone else is **at risk of harm**  we will **tell someone** in your **local healthcare services.**  **We** will tell **your GP** that you are **taking part** in the **study.** |
| 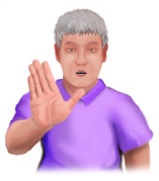 | **Contact us** if you would like your **information removed** from the **study**.  Contact us within **2 weeks**.  After 2 weeks your information **cannot** be **removed.** |


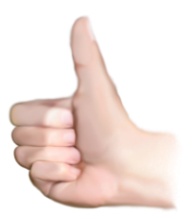
**What might be good about taking part?**

| 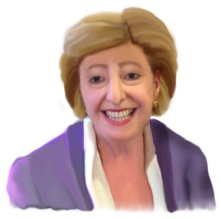 | You will **help**  people in the **future.**  They will get **better help.** |
| --- | --- |
| 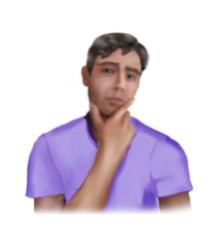 | You may **enjoy** taking part.  You may find it **interesting.** |


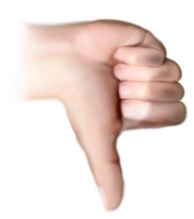


**What might be difficult about taking part?**

| 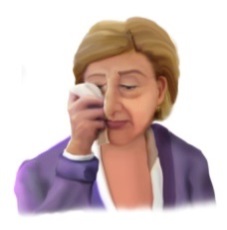 | You may find the **questions** in the questionnaires **upsetting.**  You might not **like being watched**  or **video recorded** when taking part in **therapy.** |
| --- | --- |

**Is the research safe?**

| 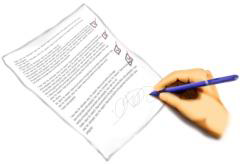  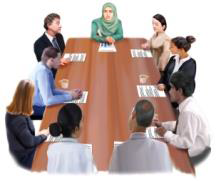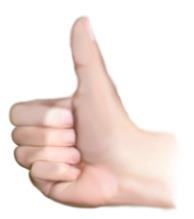 | A committee **decides if research can happen.**    This is the **Essex Research Ethics Committee** (Ref: 21/EE/0115)**.**  They say that **this research can happen.**  They say that it is **safe.**  They say that it has been **planned properly.** |
| --- | --- |

**Will I be harmed by the research?**

| 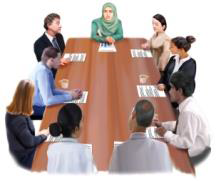 | This is very **unlikely.**  However, the NHS has set up **a committee.**  This committee will **monitor the research.**    The committee has **different people** from those who do this research. |
| --- | --- |

**What will happen after the research?**

| 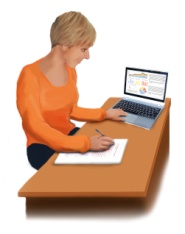 | The **researchers** will **look at the results.** |
| --- | --- |
| 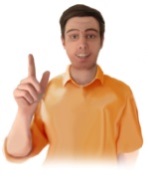 | They will **learn more** about **supporting** people with **aphasia** after **stroke.** |

**What will happen to the results?**

| 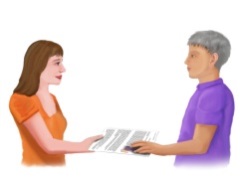 | **We** will **send** you a **summary** of the **results.** |
| --- | --- |
| 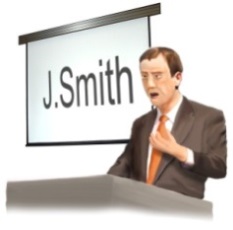 | We will share the **results** with **other researchers** at **conferences** and **meetings** in **academic journals.** |
| 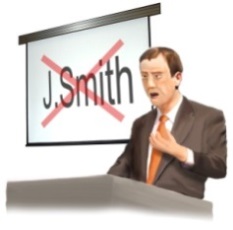 | The results will **not use your name.** |
| 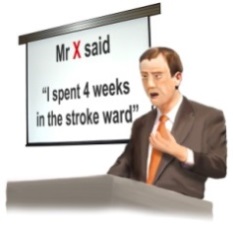 | The results may include **what you said**  but **not who said it.** |

**What next?**

| 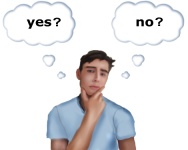 | Do you  **want** to **take part?**  **You** need to **decide.** |
| --- | --- |
| 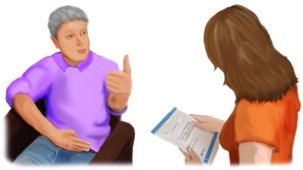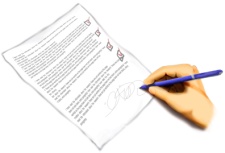 | If you want to **take part** we will ask you to sign a **consent form. Someone can help** you with this.  This says that **you understand** the research and **you agree** to take part.  We may ask you to **agree verbally** if we meet you by **videocall**. We will take a **sound recording** of this. |
| 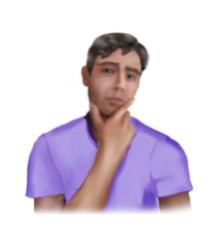 | If you decide **not** to take part you **do not need to do anything.** |
| 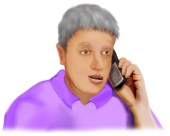  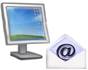 | If you want **more information**  **Please contact** the **researcher**: Faye Wray  **Telephone** 01274 38 3400 Or  **Email** [f.d.wray@leeds.ac.uk](mailto:f.d.wray@leeds.ac.uk) |

**Claims and insurance**

| 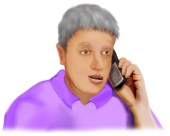  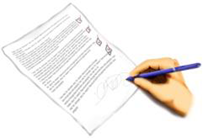 | If  you**take  part**  in  the  research  and  **if  you  think**  you  were  **harmed**  you **may be able to claim compensation** from the University of Leeds.  **You** may have to **pay legal costs.** |
| --- | --- |

**What if there is a problem?**

| 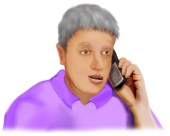  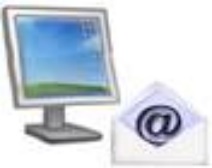 | **If you want to discuss** this study **with someone independent** contact:  **Clare Skinner** on: 0113 343 4897 or email: [governance-ethics@leeds.ac.uk](mailto:governance-ethics@leeds.ac.uk) |
| --- | --- |

**Where could I get further support?**

| 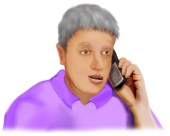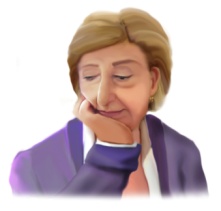 | **If** you have **concerns about the care** you have had in the NHS  or you **need advice** about  **other organisations who can help** you can contact:  **The Patient Advice and Liaison Service (PALS). Phone 111** for details of **your nearest PALS or visit** [**https://www.nhs.uk/common-health-questions/nhs-services-and-treatments/what-is-pals-patient-advice-and-liaison-service/**](https://www.nhs.uk/common-health-questions/nhs-services-and-treatments/what-is-pals-patient-advice-and-liaison-service/)  Or **the Stroke Association** on  **0303 3033 100** |
| --- | --- |

**Thank you for taking the time to read this information.**

This study is organised and run by the Academic Unit for Ageing and Stroke Research (a department of the University of Leeds) at Bradford Teaching Hospitals NHS Foundation Trust. This study is funded by The Stroke Association. The sponsor for the research is the University of Leeds. The study has been approved by East of England - Essex Research Ethics Committee (Ref: 21/EE/0115). Sources: All images in this information sheet are from: https://www.nihr.ac.uk/stroke/pcpie/enabling-people-with-aphasia-to-participate-in-research-resources-for-stroke-researchers/ [accessed 10th July 2018]
