# Supplementary material for: Protocol for a feasibility study evaluating a supported self-management intervention for stroke survivors with aphasia (StarStep study)
Source: Pilot Feasibility Stud. 2025 Jan 30;11:11. doi: 10.1186/s40814-024-01589-y (PMC11780826; doi:10.1186/s40814-024-01589-y)
Supplement: Supplementary file 3 — Additional file 3. Accessible consent form. [file 40814_2024_1589_MOESM3_ESM.docx]

| Office use only | | |
| --- | --- | --- |
| Participant ID: | Initials: | Site ID: |

Study title: Feasibility study of a supported self-management intervention for aphasia (StarStep study)

**CONSENT FORM FOR QUESTIONNAIRES AND OBSERVATIONS**


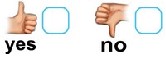
 **Please initial if possible (or tick)**

| 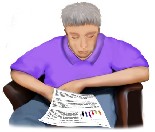 | I have **read** the **information** about the **research** | 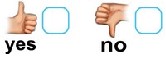 |
| --- | --- | --- |

| 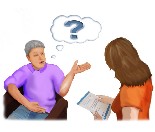 | I have had the **chance** to **ask questions** about the research | 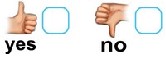 |
| --- | --- | --- |

| 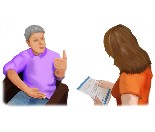 | I am **happy** with the **answers** to my questions | 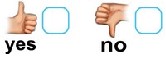 |
| --- | --- | --- |

| 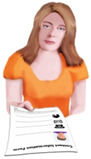 | I **agree** to answer **questions** about my **health and wellbeing** | 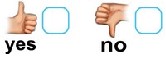 |
| --- | --- | --- |

| 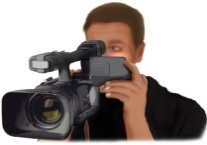 | I **agree** to my **speech and language therapy** being **video recorded** | 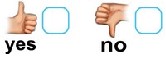 |
| --- | --- | --- |

| 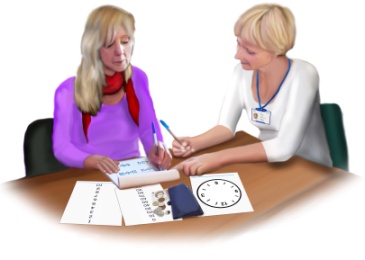 | I **agree** to a **researcher** **observing** one of my speech and language **therapy** sessions**.** | 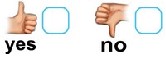 |
| --- | --- | --- |

| 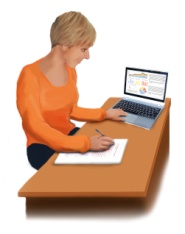 | The **researcher** will make **notes** about the **therapy** | 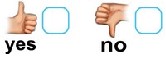 |
| --- | --- | --- |

| 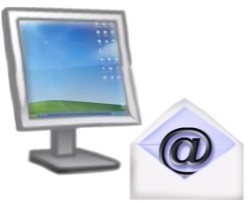 | I **agree** to a **researcher** looking at **my care records** to **collect data** about my **health** | 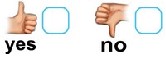 |
| --- | --- | --- |

| 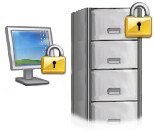 | I understand that **information** about me will be **kept safe** at the Academic Unit for Ageing and Stroke Research | 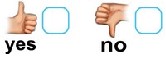 |
| --- | --- | --- |

| 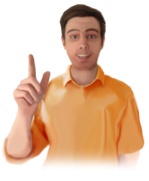 | **Information** I give may be **used** to **develop** new **research** | 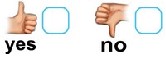 |
| --- | --- | --- |

| 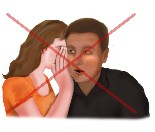 | It will **not** be **shared** with **anyone outside** **the research team unless** there are **concerns** that I or someone else is **at risk of harm** | 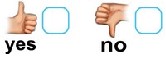 |
| --- | --- | --- |

| 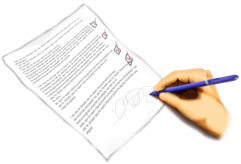 | A **copy of this form** will be **stored** at the Academic Unit for Ageing and Stroke Research | 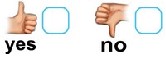 |
| --- | --- | --- |

| 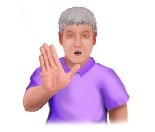 | I understand that **I can stop being in the research** at any time | 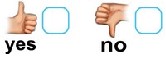 |
| --- | --- | --- |

| 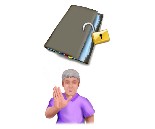 | If I **stop** being in the research **information** already collected about **me will** still be **used** | 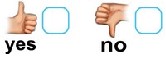 |
| --- | --- | --- |

| 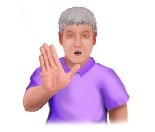 | **If I stop I do not** have to **give a reason**…and I will **still get my normal care** | 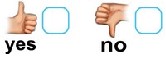 |
| --- | --- | --- |

| 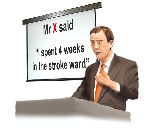 | The results may include **what** I said but **not** use my **name** | 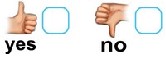 |
| --- | --- | --- |

| 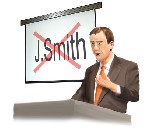 | I understand that when the **results** are shared, the researcher will **not use my name** | 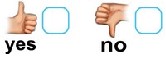 |
| --- | --- | --- |

| 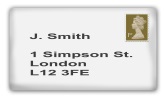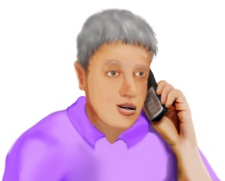 | A **researcher** may **contact** you about being **interviewed** in the **future** | 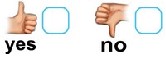 |
| --- | --- | --- |

| 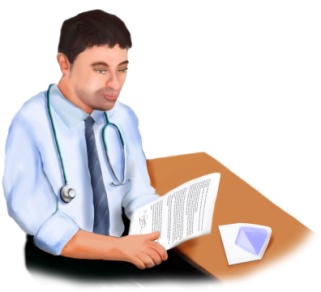 | I **agree** for my **GP** to be told that I am **taking part** in this **research** | 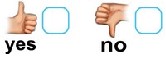 |
| --- | --- | --- |

| 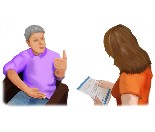 | I **agree** to take part in this **research** | 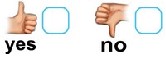 |
| --- | --- | --- |


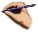

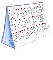
**Name _______________________________________**

**Signature ___________________ Date _________**


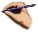

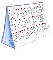
**Witness Name _______________________________________**

**Signature ___________________ Date _________**


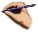

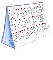
**Researcher’s Name _______________________________________**

**Signature ___________________ Date _________**
